# Evaluation of an augmented reality navigational guidance platform for percutaneous procedures in a cadaver model

**DOI:** 10.1117/1.JMI.11.6.062602

**Published:** 2024-02-15

**Authors:** Gaurav Gadodia, Michael Evans, Crew Weunski, Amy Ho, Adam Cargill, Charles Martin

**Affiliations:** a VIR Chicago, Interventional Radiology, Chicago, Illinois, United States; b MediView XR, Inc., Cleveland, Ohio, United States; c Cleveland Clinic, Diagnostic Radiology, Interventional Radiology, Cleveland, Ohio, United States

**Keywords:** augmented reality, needle guidance, percutaneous procedures, interventional radiology, visualization, ultrasound

## Abstract

**Purpose:**

The objective of this study is to review the accuracy of an augmented reality navigational guidance system designed to facilitate improved visualization, guidance, and accuracy during percutaneous needle-based procedures including biopsies and ablations.

**Approach:**

Using the HoloLens 2, the system registers and projects 3D CT-based models of segmented anatomy along with live ultrasound, fused with electromagnetically tracked instruments including ultrasound probes and needles, giving the operator comprehensive stereoscopic visualization for intraoperative planning and navigation during procedures.

Tracked needles were guided to targets implanted in a cadaveric model using the system. Image fusion registration error, the multimodality error measured as the post-registration distance between a corresponding point measured in the stereoscopic CT and tracked ultrasound coordinate systems, and target registration error, the Euclidean distance between needle tip and target after needle placement, were measured as registration and targeting accuracy metrics. A t-distribution was used for statistical analysis.

**Results:**

Three operators performed 36 total needle passes, 18 to measure image fusion registration error and 18 to measure target registration error on four targets. The average depth of each needle pass was 8.4 cm from skin to target center. Mean IFRE was 4.4 mm ($H_{0}$: μ=5  mm, P<0.05). Mean TRE was 2.3 mm ($H_{0}$: μ=5  mm, P<0.00001).

**Conclusions:**

The study demonstrated high registration and targeting accuracy of this AR navigational guidance system in percutaneous, needle-based procedures. This suggests the ability to facilitate improved clinical performance in percutaneous procedures such as ablations and biopsies.

## 1 Introduction

Minimally invasive percutaneous procedures are increasingly indicated and used in modern medicine. For example, percutaneous biopsies and thermal ablations are common in the growing field of image-guided interventional oncology.^1^^,^^2^ These procedures have been increasingly used to diagnose and treat hepatic, renal, and other soft tissue tumors, especially in patients not eligible for surgical resection.^3^^–^^6^ One drawback is that images used for guidance in these minimally invasive methods are displayed on fixed two-dimensional (2D) monitors, and some, including computed tomography (CT), expose the patient to radiation.^7^ Furthermore, targets for percutaneous procedures are often in sensitive or difficult-to-access locations and current image guidance is often not ideal due to issues with two-dimensionality.

Augmented reality (AR) may help to facilitate ideal needle trajectory planning and guidance for percutaneous procedures by allowing providers to visualize their intended target in a three-dimensional (3D) space.^8^ AR involves projection of digital content into the real world through mediums such as a head-mounted display (HMD) device. As such, the user can visualize both the real world and projected digital content at the same time.^9^

A high degree of accuracy is required for AR to be useful during image-guided needle-based procedures. Compared to traditional 2D navigation systems, measuring the accuracy of 3D navigation systems using stereoscopic projections is inherently challenging. Obtaining measurements that include depth, or the Z axis, may be influenced by the vergence accommodation.^10^

To date, few studies have evaluated the accuracy of an AR guidance system for minimally invasive procedures in soft tissue. Target registration error (TRE) is a measurement of the main system error. It is defined as the Euclidean distance between the needle tip and the target after the needle has been placed. TRE is representative of how close a user can guide their needle tip toward an identifiable location of a target, such as the center of a tumor. Image fusion registration error (IFRE) is a measurement of multimodal registration error between different imaging modalities such as CT and ultrasound (US) or US and magnetic resonance imaging. Both TRE and IFRE are quantitative measurements that reflect the accuracy of a 3D needle guidance system. This is a review of a cadaver study evaluating the TRE and IFRE of an AR platform for navigational guidance in a cadaver model.

## 2 Methods

A review of the accuracy of an AR platform (XR90, MediView XR, Inc., Cleveland, OH) was completed in a single cadaver after obtaining IRB exemption, as per institutional policy. The male cadaver’s demographic description was representative of a normal male adult without underlying health concerns (Age 47 yrs., BMI 21, Height 177.8 cm, and Weight 66 kg). The cadaveric specimen was not embalmed and did not have any health conditions that would contraindicate the use of the system (such as extreme obesity, fatty liver disease, metallic implants, or fluid accumulation). Due to the use of artificial tumor targets being implanted to measure system accuracy, specimens with pre-existing lesions such as hepatocellular carcinoma (HCC) or metastasized cancer to the liver were not considered for this evaluation.

### 2.1 System Description

The AR platform registers and projects 3D CT-based models of segmented anatomy while also displaying live US, fused with electromagnetically (EM) tracked instruments such as US probes and needles. The system is comprised of a HoloLens 2 (Microsoft, Redmond, WA) AR HMD, Aurora^®^ Electromagnetic Tracking system (NDI, Waterloo, Canada) including EM sensor-equipped components such as interventional eTRAX needle (CIVCO Medical Solutions, Coralville, IA), US probe, markers for performing registration, data streamer PC, and router. The system interfaces with a commercially available US system (Vivid iq, GE Healthcare, Chicago, IL) and streams data in real time to the client application on the headset using a local-area network.

An EM field generator is mounted underneath the patient table and creates an EM measurement volume around the subject. The EM volumetric cylinder created by the tracking system has a radius of 250 mm with a dome radius of 600 mm. The field is offset by 41 mm in height to account for the generator’s placement underneath the table. Twelve and fourteen-gauge eTRAX needles measuring 17 cm in length were used for the study. Real-time EM tracking data of EM sensor-equipped tools, including US probe and tracked needle (i.e., instrument), are sent to a data streamer PC. Tracking position and orientation data are sent to the client application on the HoloLens headset from the data streamer PC over a local-area network.

**Fig. 1:**
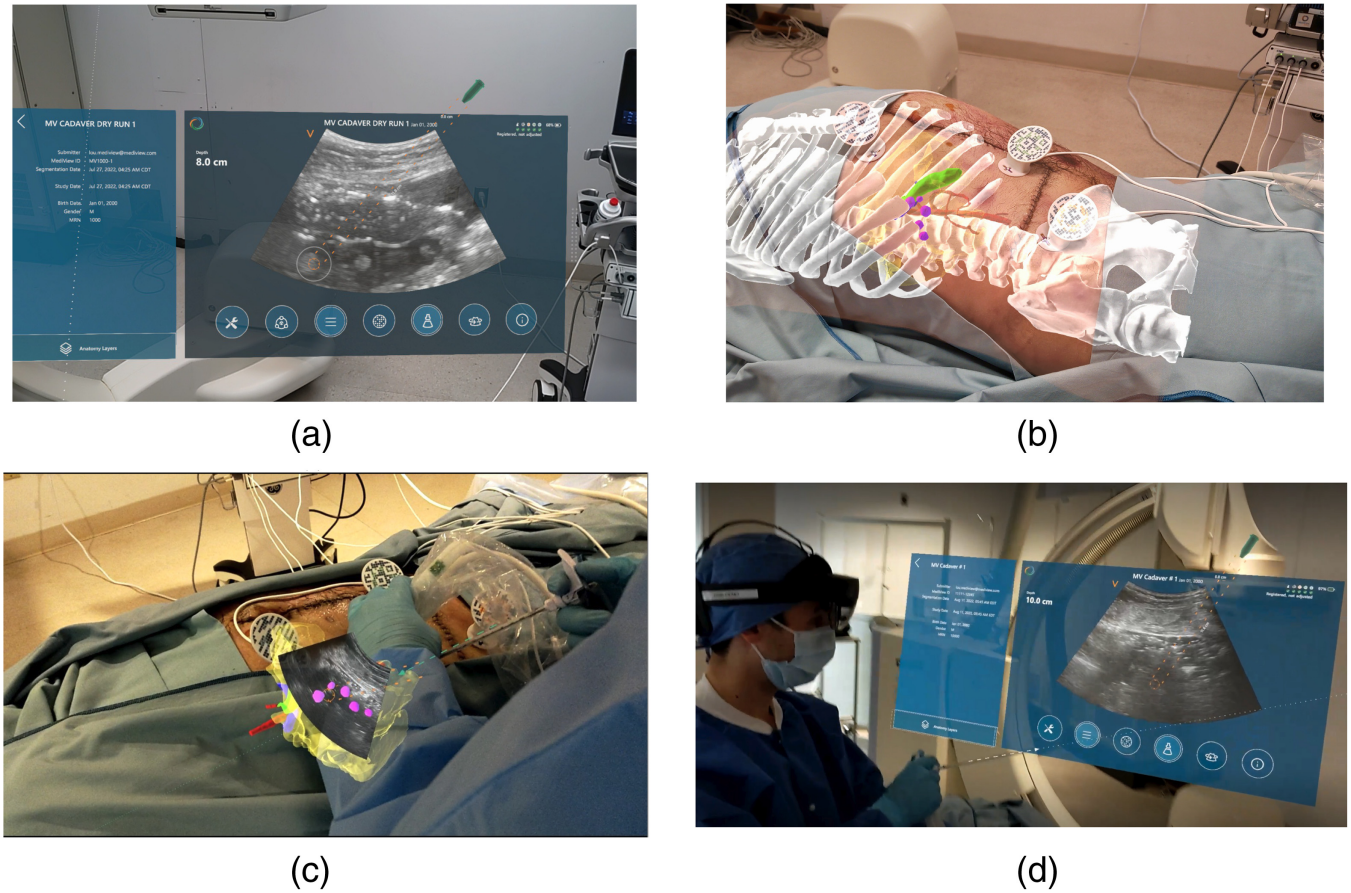
First-person views of primary system projections. (a) HUD featuring streamed US and needle guide directly from US. HUD may be positioned based on operator preference allowing for ergonomically friendly positioning. (b) Stereoscopic 3D CT anatomy registered to the subject and registered US projection. Five purple circular projections are the stereoscopic projection of implanted targets. (c) Registered US projection intersecting with stereoscopic CT targets, virtual needle trajectory (green), and EM-tracked needle with needle guide (orange). Three circular registration markers are also shown encircling the region of interest. (d) First-person view of a second operator using the HUD display to define a needle trajectory during the study. The image was taken by a user also wearing a headset so that the HUD was visible.

The system has four main types of stereoscopic projections that enable visualization, surgical guidance, and navigation. These projections include a heads-up display (HUD), which contains the main user interface and a 2D US display that may be placed in an ergonomic position for the user, as well as three projections that are registered to each other by the user. The three registered virtual objects are stereoscopically projected using sensor-equipped registration markers that are placed on the same skin-marked fiducial locations from the pre-procedural CT scan. The three registered projections include (1) 3D patient-specific models of the anatomy, implanted tumor targets, and skin fiducials segmented from pre-procedural CT data for gross localization and anatomical spatial understanding, (2) a virtual representation of the live US B-sector projected coaxially from the US probe that matches the live image on the HUD and scanner (registered US projection), and (3) a virtual representation of an EM-tracked interventional instrument’s trajectory (virtual needle trajectory). The four system components are shown in Fig. 1.

At the start of a procedure, the operators place three registration markers [see Figs. 1(c) and 1(d)] over the skin-marked fiducial locations from the preprocedural CT scan. To register the CT-based anatomical models, US projection, and virtual needle trajectory, the user initiates registration in the client software and gazes at each marker sequentially. The registration markers contain both an optical image and embedded EM sensor that transform the position and orientation of objects in their respective CT/EM coordinate systems to the common coordinate system of the headset. Due to the static nature of the 3D models based on preprocedural CT, operators are instructed to not use the CT-based stereoscopic anatomy for guidance, but rather as a supplementary visualization tool to assist with gross localization of targets under US and for 3D spatial understanding of the targets related to critical structures. The system is used as an adjunct to standard-of-care US imaging per its intended use.

### 2.2 Target Implantation and Segmentation

**Fig. 2:**
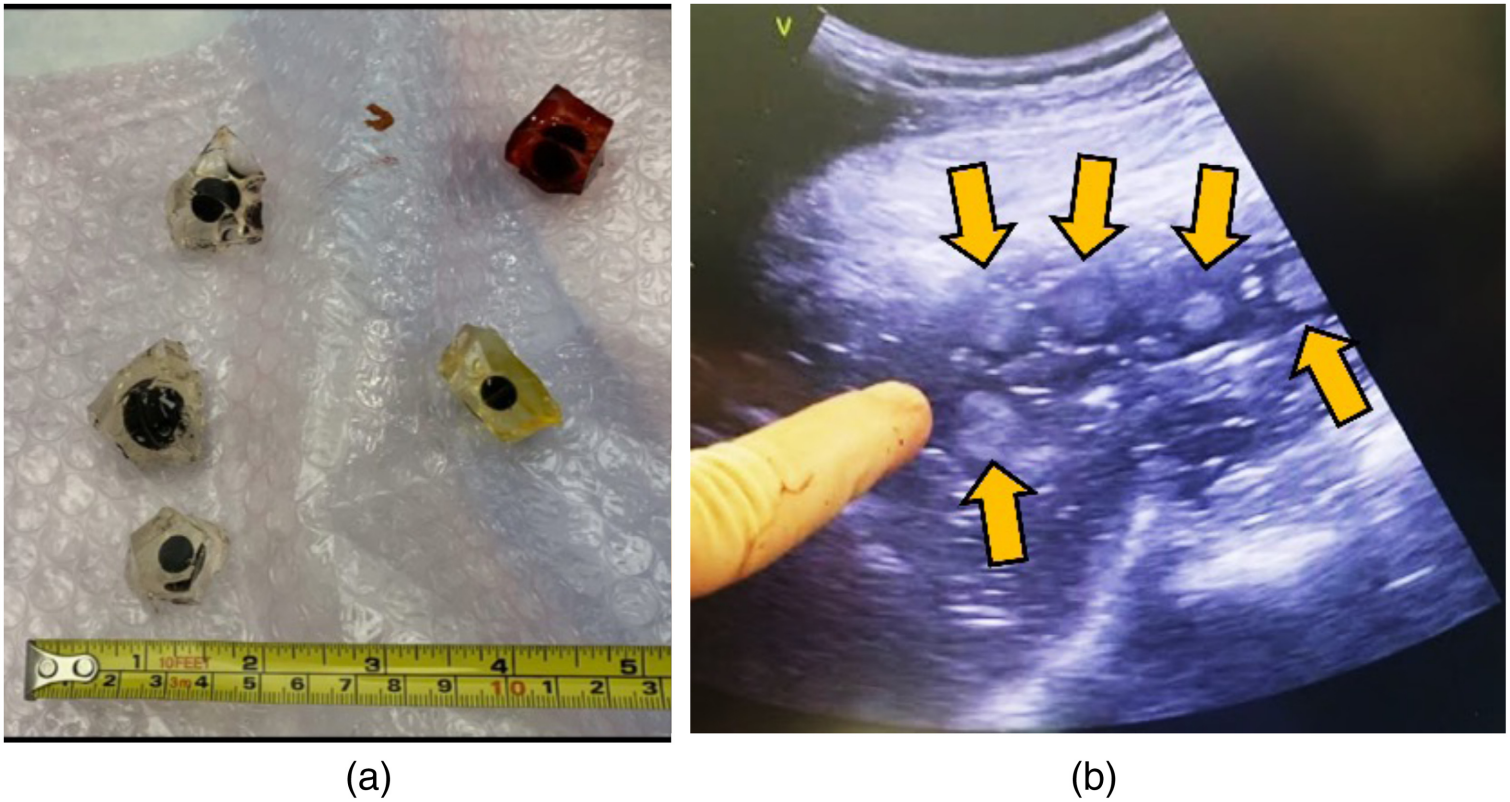
(a) Targets surrounded by nonechogenic gel before implantation. (b) Targets as seen under US after being inserted into the right lobe of the liver.

Echogenic spherical targets were surgically implanted into the liver of a cadaver abdomen specimen to simulate tumors. The spherical targets were composed of Zerdine^®^ material and surrounded by ∼1  cm of non-echogenic gel, as shown in Fig. 2. The nonechogenic gel served to help secure the targets post-implantation without distorting the visible boundary under US. The targets were implanted on the right lobe of the liver near critical structures (such as the gallbladder) to mimic physiologically challenging insertion angles for ablation. Fiducial markers were placed on the skin surface of the specimen and the specimen was imaged using CT. The resulting DICOM data was segmented into object files (OB)J files and decimated for rendering at the required frame rate of the HoloLens. Decimated OBJ segmentation data was imported into the system prior to the procedure.

### 2.3 Sample Size

A preliminary study was used to justify a sufficient sample size, in which the TRE sample mean was 3.2 mm with standard deviation of 0.9 mm (n=6), and the IFRE sample mean was 2.5 mm (N=8) with a standard deviation of 0.5 mm. Per this preliminary evaluation, a study with a detectable effect size of 0.8 mm (mean difference 5.0−4.2  mm), an estimated standard deviation of 1 mm, and a power of 90% required a total sample of at least 15 procedures to test the mean TRE at 5% level using a one-tailed test, with TRE requiring larger sample size than IFRE to demonstrate statistical significance based on preliminary evaluation.

### 2.4 Target and Operator Selection

Three nonclinical operators familiar with the technical characteristics and functions of the platform performed the procedures. Prior to performing procedures on the cadaver, each operator successfully completed execution of in-plane and out-of-plane needle approaches on a phantom using the system, as well as training on the TRE and IFRE measurement methods. To satisfy power requirements (n = at least 15 per metric), and assuming each operator would perform two passes on each target, five spherical targets were implanted in the liver of a single cadaver. The target selection order was randomized for each operator using a random number generator.

Generally, nodules need to be at least 1 cm in diameter for evaluation or intervention to take place.^11^^,^^12^ A null hypothesis of ≤5  mm allows for the assessment of accurate needle placement within the radius of a tumor with a diameter of at least 1 cm. As such, we hypothesized that the mean of both TRE and IFRE would be statistically significant <5  mm and strove for an average depth of ∼7  cm to simulate real-world clinical application.

### 2.5 Accuracy Metrics

The accuracy of the system was evaluated using TRE and IFRE. TRE is a measurement of total aggregate system error and is a standard accuracy metric used in image-guided systems to describe the Euclidean distance between two registered virtual objects.^13^ In this study, TRE was measured to report the post-registration Euclidean distance between the tip of the virtual needle trajectory and the center of the target imaged under the real-time registered US projection, in accordance with the intended use of the device. TRE was computed as the Euclidean distance between the tip of the needle, $P_{\text{tip}}$, and the center of the target, $P_{\mathrm{ctr}}$, after needle placement as measured with US by localization on the US using the HUD. The distance is computed as

$$\mathrm{TRE}=\sqrt{{\left(P_{\text{tip},x}-P_{\mathrm{ctr},\mathrm{US},x}\right)}^{2}+{\left(P_{\text{tip},y}-P_{\mathrm{ctr},\mathrm{US},y}\right)}^{2}+{\left(P_{\text{tip},z}-P_{\mathrm{ctr},\mathrm{US},z}\right)}^{2}.}$$ (1)

IFRE was measured to report the post-registration Euclidean distance between the registered US projection and CT-based stereoscopic anatomy. IFRE was measured after initial system registration and CT adjustment. In this method, the operator translates the CT-based Anatomy based on corresponding points from the target located on the CT-based projections and US HUD. IFRE is computed as the post-registration Euclidean distance between the center of the target, $P_{\mathrm{ctr}}$, as measured in CT coordinates and measured on the US using the HUD, $P_{\mathrm{ctr},\mathrm{US}}$. The distance is computed as

$$\mathrm{IFRE}=\sqrt{{\left(P_{\mathrm{ctr},\mathrm{CT},x}-P_{\mathrm{ctr},\mathrm{US},x}\right)}^{2}+{\left(P_{\mathrm{ctr},\mathrm{CT},y}-P_{\mathrm{ctr},\mathrm{US},y}\right)}^{2}+{\left(P_{\mathrm{ctr},\mathrm{CT},z}-P_{\mathrm{ctr},\mathrm{US},z}\right)}^{2}.}$$ (2)

### 2.6 Simulated Procedure

The system was set up in an interventional suite prior to the procedure. The cadaveric specimen was placed on the table in the supine position with the region of interest approximately centered in the EM field generator. Registration of the CT and EM coordinate space was performed by placing the registration markers at the skin-marked locations from the pre-procedural CT. The registration markers contain both an optical image pattern and an EM sensor that enable registration to the common headset coordinate system. Once registered, each operator used the CT-based anatomy for gross localization of targets under US. The registered US projection in conjunction with the virtual needle trajectory was used for pre-insertion trajectory planning. Once planning was completed the operators navigated to the center target using the EM-tracked needle.

To measure TRE, the tip of the virtual needle trajectory was placed at the center of the spherical target imaged on the registered US projection image using the XR90 system (in conjunction with standard of care) for guidance and navigation (including critical structure avoidance). After needle placement, the needle was stabilized and the system was used to mark the tip of the needle as imaged under US, as well as the center of the target. Based on the 3D Cartesian point locations in the head-mounted display coordinates, the system calculated and reported a TRE measurement. After measuring TRE, the operator held the needle at the point closest to the skin (percutaneous access point) insertion point and withdrew the needle. The needle depth for each placement was measured using calibrated calipers to measure the tip of the needle to the percutaneous access point.

To measure IFRE, the distance between corresponding points in the CT and US coordinate systems was minimized using a registration adjustment that allows the operator to translate the CT-based anatomy to match a corresponding location indicated on the live US. Once the distance between corresponding virtual points was minimized, the operators used the HUD to mark the center of each simulated target. Using a voice command, the AR platform calculated and reported an IFRE using the post-registration Euclidean distance between the center of the target marked on the US image and the center of each target from the CT coordinate system.

## 3 Results

All procedures occurred during August 2022. A procedure was defined as a single attempt to reach the center of the spherical implanted target using AR guidance in adjunct with US. As noted above five targets were implanted into the cadaveric liver. These ranged in diameter from 11.4 to 13.8 mm and were implanted at an average depth of 6.3 cm below the skin of the model. Descriptive data of the targets is presented in Table 1. Of note, one target was not visible under US immediately and so was not used for procedures. As such, data collected from 36 procedures performed by three users on four targets over 2 days within a single cadaver was reviewed.

**Table 1:** Target descriptions.

Target	Target diameter^14^	US depth (cm)	Target sufficiently visible post-implantation surgery (yes or no)
1	11.4	5.82	Yes
2	13.1	6.18	Yes
3	13.8	5.73	Yes
4	13.8	7.69	Yes
5	Excluded	5.99	No

**Fig. 3:**
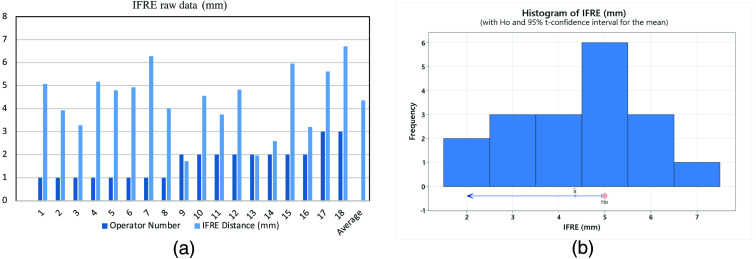
(a) IFRE raw data in mm and (b) histogram of image IFRE.

**Fig. 4:**
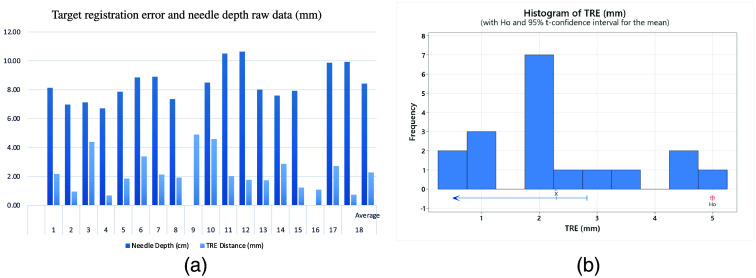
(a) TRE, first pass, needle gauge, and needle depth raw data (b) histogram of TRE.

On day one, IFRE measurements were collected on the four visible targets. Two operators performed two passes on each target (n=4) for a total of 16 passes. To satisfy IFRE power requirements, A third operator performed two additional passes on a single target for a total of 18 procedures. After day one of procedures an additional target was no longer visible due to tissue decomposition and related cephalad excursion of the liver. On day two, TRE measurements were collected on the three remaining visible targets. Three operators each completed a total of two procedures on three different targets for a total of 18 procedures evaluating TRE. Data for IFRE metrics can be found in Fig. 3 raw data for TRE, and Needle Depth can be found in Fig. 4.

Needle depth ranged from 6.7 to 10.6 cm with a mean of 8.4 cm All users were able to reach the target on the first attempt for every procedure. The mean result for TRE was 2.3 mm with a 95% upper bound of 2.9 mm. A T-distribution sample resulted in a statistically significant TRE of <5  mm (n=18; P<0.00001). The mean result for IFRE was 4.4 mm with a 95% upper bound of 4.9 mm. A t-distribution sample resulted in a statistically significant IFRE of <5  mm (n=18; P<0.05). Cumulative T distribution statistics for IFRE and TRE can be found in Table 2.

**Table 2:** One-sample T-test for IFRE and TRE.

Sample	N	Mean	StDev	Se Mean	95% Upper Bound for μ
IFRE (mm)	18	4.4	1.4	0.3	4.9
TRE (mm)	18	2.2	1.3	0.3	2.8
*μ: population mean of IFRE, TRE*					
**Test**					
Null hypothesis			$H_{0}$: μ=5 mm		
Alternative hypothesis			$H_{1}$: μ<5 mm		
**Sample**		**T Value**		**P-Value**	
IFRE		−1.93		0.035	
TRE		−8.85		0.000000045	

## 4 Discussion

This study demonstrates the accuracy of an AR system for percutaneous needle guidance in a cadaver model when used in adjunct to standard-of-care imaging. As stated, minimally invasive percutaneous needle-based procedures such as biopsies, thermal ablations, and drains are increasingly being used for diagnosis and treatment in multiple organ systems.^3^^–^^6^ Sub-centimeter accuracy is required for these procedures to have clinical benefits for patients.^12^^,^^14^^–^^16^ A primary factor limiting percutaneous therapies is the quality and nature of image guidance currently available to operators, making the required accuracy challenging to achieve.^17^ The standard of care image guidance utilized for these procedures, primarily US and CT, are limited by two-dimensional projections of inherently complex 3D anatomy, and, for the latter, the need for ionizing radiation. Surgical navigation systems aim to improve image guidance and have been shown to improve percutaneous needle-based procedures by improving targeting accuracy and decreasing the number of intra-procedural CT scans required to achieve correct placement.^18^^,^^19^ Multimodal image fusion platforms can help improve depth perception and spatial anatomic understanding.^20^^,^^21^ Head-mounted-display-based multimodal image fusion AR platforms such as the one in this study provide improved depth perception and spatial understanding, while also allowing true three-dimensional and even interactive projections.^22^^,^^23^ Overall, the use of AR for surgical navigation may potentially improve operator confidence and facilitate percutaneous procedures on more challenging targets that would not be appreciable with only standard-of-care guidance.^9^^,^^23^^–^^25^

Furthermore, the HMD-based AR environment allows for image displays to be projected in places of the operator’s choosing, including locations that are more ergonomically friendly such as directly in front of their hands and on the operative site itself. For instance, in this platform, the live-streamed US display projected on the HUD can be positioned anywhere, while the stereoscopic projections of the CT-based anatomy and EM-tracked virtual needle trajectory remain registered to the patient. This has implications for room positioning and workflow, comfort, ergonomics, and related workplace injuries with implications for disability.^26^^–^^28^

Of note, many of the above benefits have been demonstrated in work by other groups on other platforms, and in earlier benchtop and clinical usability studies of the same platform used in this study.^22^^,^^23^ However, no prior study on this platform has evaluated intraoperative accuracy, and none of the above benefits would be clinically applicable without high image fusion registration fidelity or targeting accuracy.

In this cadaveric study, the statistically significant TRE provides evidence that this AR needle guidance system can be used to reach targets with the precise degree of accuracy required for clinical applications. A mean TRE<5  mm demonstrates that this AR needle guidance system has the potential to be used for targeting within the boundaries of the smallest operable tumors. The statistically significant IFRE<5  mm also suggests that registration between the stereoscopic projection 3D CT anatomy and the registered US projection is reliable for the accurate localization of a target.

### 4.1 Limitations

There are limitations in terms of the clinical applicability of the findings in this study. This sample size was limited to a single cadaver model, a single center, and three operators. While this study was adequately powered and yielded statistically significant results, a larger sample would facilitate a stronger study conclusion.

Moreover, a cadaveric model is not an ideal model to assess *in vivo* clinical applicability and accuracy. A contrast-enhanced pre-procedural CT could not be obtained due to lack of blood flow, preventing vessel visualization and optimal tumor and organ delineation. Furthermore, due to lung compression and tissue dehydration, the model experienced cephalad excursion of the liver into the thorax, causing challenges with US visibility between the ribs and ultimate loss of ability to view certain targets under US as described above. Most importantly, breathing, and gross patient motion could not be simulated, which are major complicating factors in all image-guided percutaneous procedures.^29^

Of note in this regard, the current platform is intended to be used as an adjunct, with the projected segmented CT anatomy providing an understanding of spatial anatomy, while the real-time streaming US imaging and EM-tracked US probe and needle are used for trajectory planning and needle guidance, which obviates some of these limitations. However, multicenter studies comparing the AR platform to standard of care US- and CT-based guidance in live subjects with inherent motion would further evaluate system accuracy in a setting more representative of a clinical environment.

## 5 Conclusion

This study demonstrates the targeting and registration accuracy of this multimodality AR image guidance platform in percutaneous needle-based procedures in a cadaveric model. These results point to the potential clinical utility of this platform as an AR solution to improve clinical performance in percutaneous procedures such as biopsies and thermal ablations, warranting further *in vivo* evaluation.

## Data Availability

All data in support of the findings of this paper are available within the article. Descriptive data is found in Table 1. Raw data is included in Tables 1 and 2.
